# Research progress on the epidemiology and diagnostic technologies of urinary tract infections in kidney transplant recipients

**DOI:** 10.3389/fimmu.2026.1913840

**Published:** 2026-10-30

**Authors:** Liqi Fan, Yiming Zhou, Bin Shen, Yunheng Zhou

**Affiliations:** 1 Department of Clinical Laboratory, Zhabei Central Hospital of Jing’an District, Shanghai, China; 2 Fuyang Medical School, Fuyang Normal University, Fuyang, Anhui, China

**Keywords:** kidney transplantation, laboratory testing, post-kidney transplantation urinary tract infections, post-transplant infection, transplant immunology

## Abstract

In recent years, the increase in poor lifestyle and dietary habits (such as smoking, unhealthy eating) coupled with the high prevalence of chronic comorbidities including diabetes and hypertension has led to a rising number of patients with kidney disease. Kidney transplantation (KT), as an important means to improve the quality of life for patients with end-stage kidney disease (ESKD), has been widely applied in clinical treatment. However, post-kidney transplantation urinary tract infections (Post-KT UTIs), as a serious postoperative complication, severely threatens the safety of transplant recipients and the long-term function of grafts. Post-KT UTIs refer to an inflammatory reaction resulting from pathogen proliferation in the urinary system after transplantation, which may include cystitis, pyelonephritis, and in severe instances, can advance to bacteremia or sepsis, endangering both patient and graft survival. Post-KT UTIs are commonly caused by Gram-negative bacterial infections, notably by species such as Klebsiella pneumoniae and Escherichia coli (E. coli). Currently, the clinical detection and treatment of Post-KT UTIs remain based primarily on traditional urine culture and empirical antimicrobial therapy. This review introduces advanced detection methods such as next-generation sequencing and clustered regularly interspaced short palindromic repeats (CRISPR), aiming to offer new strategies for diagnosing Post-KT UTIs. Moreover, it reviews recent advancements in the epidemiology, treatment methods, and preventive strategies of Post-KT UTIs, and the role of gut and urinary microbiota in infection development, aiming to facilitate the development of novel therapeutic agents, vaccine formulations, and refined treatment strategies for precise management of Post-KT UTIs.

## 1 Introduction

Recently, the number of kidney disease patients has risen concurrently with the increase in unhealthy lifestyle and dietary habits (e.g., smoking, unhealthy eating, and physical inactivity) and the high incidence of chronic comorbidities such as diabetes and hypertension (1–3). With the advancement of kidney transplantation (KT) and the precise application of immunosuppressive therapy, along with continuous optimization of pre- and post-operative management, the quality of life and survival rates of many patients with end-stage kidney disease (ESKD) have significantly improved. However, postoperative immunosuppressive therapy renders patients susceptible to various infections, among which Post-KT UTIs primarily caused by Gram-negative bacterial infections are one of the main complications affecting graft survival and patient quality of life (4). The intricate spectrum of pathogens involved in Post-KT UTIs, their atypical clinical presentations, and the specificities of diagnostic and therapeutic interventions establish Post-KT UTIs as a crucial subject requiring thorough investigation in the transplantation domain. Given the critical impact of Post-KT UTIs on kidney allograft and patient outcomes, this review synthesizes current research, with special emphasis on the expanding spectrum of causative pathogens—including the rising significance of viruses, such as BK polyomavirus (BKV), JC polyomavirus (JCV), cytomegalovirus (CMV), and adenovirus (ADV), mycoplasmas (*Ureaplasma* spp. and *Mycoplasma hominis*), and multidrug-resistant bacteria/fungi—alongside the transformative potential of next-generation diagnostics such as metagenomic next-generation sequencing (mNGS) and clustered regularly interspaced short palindromic repeats (CRISPR)-based assays (e.g., LbuCas13a, ERA/CRISPR-Cas12a). We review the evolving pathogen spectrum and emerging diagnostic technologies, thereby offering a theoretical basis and possible future directions for precision management.

## 2 Epidemiology of post-KT UTIs

KT represents the treatment of choice for patients with ESKD. Nonetheless, the administration of immunosuppressive agents post-surgery has resulted in infections being a prevalent complication associated with KT (5). The urinary tract is a common site for infections following KT. A meta-analysis of 13 studies involving 3,364 kidney transplant recipients reported a pooled UTI prevalence of 38% (95% CI 29%–47%) (6).

In clinical settings, the post-operative course following KT is typically divided into three phases: early (within 1 month post-surgery), mid-term (2 to 6 months post-surgery), and long-term (follow-up beyond 6 months) (7). Although Post-KT UTIs can occur at any stage following KT, studies have reported that there are distinct temporal distribution characteristics associated with the incidence of Post-KT UTIs, with a higher incidence in the early post-transplant period. Furthermore, the incidence shows a stepwise decrease over time post-surgery (8).

Post-KT UTIs are influenced by multiple preoperative, intraoperative, and postoperative factors, summarized in **Table 1** (9–27). Established risk factors include female sex, advanced age, comorbidities such as diabetes, deceased donor grafts, indwelling catheters, and immunosuppressive therapy. Emerging associations include electrolyte disturbances (e.g., hypomagnesemia (25)) and microbiota imbalance (28–30).

**Table 1:** Risk factors of KT UTI.

Classification	Risk factors	Risk description	Sources
Preoperative factors	Gender	Female	(8–10)
	Age	The older the patient is, the more likely the kidney transplant UTI is to occur	(4, 8)
	Disease	Diabetes; Benign prostatic hyperplasia; Giant ureteral disease; Autosomal dominant polycystic kidney disease; Recurrent UTI before transplantation, etc.	(11, 12)
Intraoperative factors	Donor type	Deceased-donor graft	(13–15)
Postoperative factors	Indwelling devices	Double-J ureteral stent placement; Prolonged urinary catheterization	(16–21)
	Immunosuppressants	Immune inhibitors such as calcineurin inhibitors (CNIs)	(10)
	Invasive urology surgery	Percutaneous renal puncture fistula; Balloon expansion, etc.	(22, 23)
	Drug-resistant bacterial infection	extended-spectrum β-lactamase (ESBL)-producing Enterobacteriaceae; multidrug-resistant Pseudomonas aeruginosa; drug-resistant Acinetobacter baumannii, etc.	(24)
	Electrolyte disturbances	Hypomagnesemia	(25)

Furthermore, the introduction of novel therapeutic modalities has also created new potential sources of infection risk. Sodium-glucose cotransporter 2 (SGLT2) inhibitors (e.g., dapagliflozin, empagliflozin) are increasingly used in diabetic kidney disease and ESKD patients pre- and post-KT (31, 32). Mechanistically, SGLT2 inhibitors increase urinary glucose excretion, which could theoretically elevate UTI risk. Nevertheless, in a study of kidney transplant recipients with diabetes, SGLT2i therapy significantly reduced HbA1c, body weight, blood pressure, and urinary protein excretion over 6–12 months of treatment (31), which may reduce the risk of developing UTI. Given the established cardiorenal protection of SGLT2i in non-transplant populations, their use in KT recipients is likely to expand. However, current observational data are limited and inconsistent, so clinicians should consider patient-specific risk factors when prescribing these agents.

In summary, Post-KT UTIs result from multiple combined risk factors rather than a single cause. Accurately managing Post-KT UTIs therefore remains a major challenge.

## 3 Mechanisms of post-KT UTIs

The occurrence and development of Post-KT UTIs is a complex process driven by the interaction between impaired host immune defense, pathogenic invasive virulence, and altered urinary tract physiological and anatomical microenvironment (16–25, 33, 34). This multi-factor interaction is the core cause of the high incidence and recurrence of Post-KT UTIs in kidney transplant recipients, and also the key reason for the difficulty in clinical treatment (35). In the following sections, we first describe how maintenance immunosuppression compromises host defense against urinary pathogens, and then delineate the pathogenic mechanisms of bacterial UTI pathogens and other post-transplant urinary and graft-associated infections.

### 3.1 Immunosuppression and impaired antibacterial host defense

Immunosuppressive therapy contributes to infection susceptibility after kidney transplantation, alongside urinary tract abnormalities, indwelling devices, and recipient comorbidities (36, 37). Experimental studies indicate that calcineurin inhibitors can impair innate antibacterial defenses in addition to their effects on adaptive immunity (33, 34).

In a murine UTI model, tacrolimus increased bladder and kidney bacterial burdens and impaired granulocyte phagocytosis and myeloperoxidase release. Reduced Toll-like receptor 5 expression in bladder macrophages provided further evidence of altered innate immune responses (33). Cyclosporine A impaired nucleotide-binding oligomerization domain-containing protein 1 (NOD1)​-mediated antibacterial defenses in experimental pyelonephritis; reduced NOD1 expression and neutrophil phagocytosis were also observed in treated renal transplant recipients (34). Together, these studies link calcineurin inhibition to impaired innate antibacterial defense in the urinary tract (33, 34). This renders calcineurin inhibition a “double-edged sword” for kidney transplant recipients, in that it suppresses rejection while simultaneously compromising the recipient’s defense against pathogens.

### 3.2 Invasion and virulence mechanisms of bacterial UTI pathogens

According to existing research reports, although the spectrum of prevalent uropathogens varies across countries and regions, E. coli is the most common bacterial pathogen in Post-KT UTIs (4, 9, 38). In addition, Gram-negative bacteria account for the majority of infections, with opportunistic pathogens including Klebsiella pneumoniae and Pseudomonas aeruginosa forming the predominant pathogenic spectrum (**Table 2**) (39).

**Table 2:** Types of pathogens causing post-kidney transplant infections.

Pathogen types	Pathogen names	Sources
Gram-negative Bacteria	*Klebsiella pneumoniae*; *E. coli*; *Pseudomonas aeruginosa*	(38, 39)
Gram-positive Bacteria	*Enterococcus faecium*; *Staphylococcus haemolyticus*	(40, 41)
Fungi	*Candida glabrata*; *Candida tropicalis*	(40, 41)
Viruses (urinary or graft-associated infections)	*BKV*; *JCV*; *CMV*; *ADV*	(42, 43)
Fastidious genitourinary organisms	*Ureaplasma urealyticum*; *Mycoplasma hominis*	(43–45)

In addition to the aforementioned Gram-negative opportunistic pathogens, mixed infections with Gram-positive bacteria represented by *Enterococcus faecium* and fungi dominated by *Candida glabrata* are increasingly common in clinical practice. These pathogens often exhibit high drug resistance phenotypes (40). Gram-positive bacteria and fungi readily form biofilms on urothelial surfaces or indwelling catheters. This biofilm structure protects pathogens from antibiotics and immune cells, leading to persistent infection and drug resistance (41).

### 3.3 Other post-transplant urinary and graft-associated infections

Beyond bacterial post-KT UTIs, viral infections also represent important complications after kidney transplantation. Together with fastidious organisms, these viruses can cause urinary and graft-associated infections in kidney transplant recipients, although their frequency and clinical significance vary according to recipient age, immunosuppressive regimen, and viral surveillance strategy. BKV and JCV primarily cause viruria and polyomavirus-associated disease (46); CMV causes systemic or graft disease (47); and ADV can cause hemorrhagic cystitis or graft dysfunction (42). *Ureaplasma species* and *Mycoplasma hominis* cause genitourinary infection that may escape routine culture (44).

Both BKV and JCV are characterized by high seroprevalence, prolonged latency, and the capacity for asymptomatic reactivation, and can persist latently in healthy individuals for extended periods. Under immunosuppression, the viruses reactivate and replicate extensively, preferentially infecting the renal tubular epithelial and urothelial cells of the allograft. This replication causes damage to the renal parenchyma and urinary tract mucosa, triggering inflammatory responses (48). Although BKV and JCV belong to the Polyomaviridae family and share approximately 75% genomic homology, the two viruses exhibit subtle differences in their pathogenic mechanisms.

BKV-associated nephropathy (BKVAN) links antiviral immunity, immunosuppressive treatment, and renal allograft injury (46, 49). BKV-specific CD4+ and CD8+ T-cell responses contribute to viral control, and recovery of these responses accompanies clearance of DNAemia. At the same time, exposure to the calcineurin inhibitor tacrolimus and elevated corticosteroid levels during immunosuppressive therapy further increase the risk of BKVAN after kidney transplantation (46). Therefore, in clinical practice, treatment strategies must integrate the recipient’s immunological risk, viral load, and graft function to strike a balance between antiviral defense and the control of alloimmune responses, thereby linking BKV disease to the broader immunological management of kidney transplantation.

Primary JCV infection usually occurs in childhood, after which the virus persists in renal tubular epithelium, the gastrointestinal tract, and the central nervous system. JCV enters cells through VP1 capsid protein binding to sialic acid-containing receptors on the cell surface (50). JCV viruria is transient with low viral load, and viremia is rare (approximately 14.2%) (50).

Unlike the aforementioned viruses, CMV is an important opportunistic pathogen after kidney transplantation and can cause systemic or tissue-invasive disease (51, 52). Infection sources include donor transmission (D+/R- high-risk combination), recipient latent virus reactivation, or community-acquired infection (51). Immunosuppressive therapy significantly increases the risk of CMV reactivation (47). CMV damages the transplanted kidney through two mechanisms. First, direct infection of renal tubular epithelial, glomerular endothelial, and interstitial cells causes cytomegaly, intranuclear inclusions (“owl’s eye” appearance), and cell necrosis (52). Second, indirect immune-mediated injury: CMV infection aggravates allograft injury through multiple mechanisms, including upregulation of human leukocyte antigen (HLA) class II molecule expression, enhancement of immunogenicity, promotion of interleukin-6 (IL-6) and tumor necrosis factor-α (TNF-α) release, and exacerbation of interstitial inflammation (47).

Besides viruses, some small non-bacterial pathogens, such as *Ureaplasma species* and *Mycoplasma hominis* can cause genitourinary tract infection and may represent either colonization or clinically relevant disease (45, 53, 54). Current mainstream detection techniques struggle to clarify the true clinical significance of a positive result. *Ureaplasma urealyticum*, as the smallest cell wall-free prokaryote, is a common commensal of the human genital tract (54). It can directly adhere to urothelial cell surfaces, acquire nutrients from host cells, and produce virulence factors including multiple banded antigen (MBA), phospholipases A and C, and GrpE protein (54). These factors induce Toll-like receptor 2 (TLR2)-mediated proinflammatory responses (55, 56).

As intracellular pathogens, *Ureaplasma* species can evade killing by cell wall-targeting antibiotics and phagocytosis by immune cells. They employ multiple immune evasion strategies: MBA undergoes size variation and phase variation through slipped-strand mispairing and DNA inversion, allowing antigenic variation to escape adaptive immune recognition; complement resistance is achieved through expression of complement regulatory factors and proteases that inactivate complement components (57, 58). Therefore, *Ureaplasma species* readily form chronic persistent infections (59). Increasing evidence indicates that they can cause severe and even life-threatening infections in immunocompromised hosts (60, 61), such as producing large amounts of urease leading to increased blood ammonia, causing seizures and cerebral edema in patients (61–63).

### 3.4 Abnormal urinary tract anatomy and physicochemical microenvironment

Intraoperative indwelling of double-J ureteral stents and long-term catheterization increase post-KT UTI risk by providing surfaces for microbial colonization and biofilm formation (16–21). The indwelling devices not only damage the urinary tract mucosal barrier during placement and removal, but also become the main site for pathogen colonization (19, 20). Pathogens can form biofilms on the surface of the devices, and the catheter-mucosa interface provides a channel for the upward migration of pathogens (21). In addition, KT leads to the abnormal anatomical structure of the urinary tract such as ureteral anastomosis, which causes urine stasis and reduces the flushing effect of urine on pathogens, further promoting pathogen proliferation (64). Hypomagnesemia, a common electrolyte disturbance in KT recipients, can impair the tight junction function of uroepithelial cells, reducing mucosal barrier stability. This, combined with therapy-induced changes in urine composition, alters the urinary microbial microenvironment and creates a favorable condition for pathogen growth and reproduction (65).

### 3.5 Gut-urinary microbiota axis-mediated pathogenic mechanisms

Longitudinal transplant studies identify the intestine as an important reservoir for urinary pathogens (28–30). In other words, the gut may serve as a source of urinary pathogens in post-KT UTIs. The gut-urinary microbiota axis is a bidirectional regulatory network connecting intestinal and urinary microecology via immune regulation, metabolic regulation and microbiota translocation pathways (28, 66). Perioperative antibiotics, immunosuppressants and surgical stress in KT recipients induce severe intestinal and urinary microbiota dysbiosis, leading to the dysfunction of this axis (66), which is an important mechanism for the occurrence and recurrence of Post-KT UTIs. In the immune regulation pathway, the decrease in bioactive metabolites (e.g., short-chain fatty acids (SCFAs)) secreted by intestinal beneficial bacteria (67) and the immunosuppressive effect of calcineurin inhibitors (66)jointly weaken mucosal immune defense, while intestinal mucosal barrier damage triggers systemic low-grade inflammation, forming a vicious circle of immune impairment and flora imbalance (68). In the metabolic regulation pathway, intestinal microbiota imbalance causes metabolic abnormalities such as hypomagnesemia and elevated urine glucose, which damage the urinary mucosal barrier and alter the physicochemical microenvironment of urine, reducing the urinary tract colonization resistance (25, 33). Longitudinal studies provide direct support for this pathway: in a cohort of 168 kidney transplant recipients, a relative intestinal abundance of ≥1% for *Escherichia* or *Enterococcus* independently predicted subsequent bacteriuria and UTI caused by the corresponding organism, and strain-level metagenomic analyses confirmed that urinary isolates originated from the gut (28). Conversely, higher fecal abundance of SCFA-producing genera (e.g., *Faecalibacterium*) was associated with a lower risk of Enterobacteriaceae UTI (67). In the microbiota translocation pathway, the overgrowth of intestinal-derived uropathogens and the damaged intestinal mucosal barrier enable pathogens to translocate to the urinary tract through hematogenous spread and fecal-perineal-urinary tract direct contact (28), and the biofilms on indwelling urinary devices further accelerate the colonization and proliferation of these translocated pathogens in the urinary tract (19–21). These findings suggest that gut microbiota composition may serve as a predictive biomarker for post-KT UTI and a target for intervention (e.g., fiber supplementation, probiotics, or fecal microbiota transplantation, the latter reported in case series for recurrent UTI) (67). However, antibiotic treatment of UTI itself profoundly disrupts the gut microbiota and current evidence is limited to small observational cohorts, therefore precluding firm conclusions until interventional studies are available.

## 4 Clinical presentations of post-KT UTIs

Clinical presentations of post-KT UTIs are diverse and often nonspecific, ranging from symptomatic lower urinary tract infection to pyelonephritis and, in severe cases, to disseminated complications such as bacteremia and urosepsis. Distinct from UTI, asymptomatic bacteriuria (ASB) is defined by significant bacteriuria without urinary symptoms or signs. It is highly prevalent in kidney transplant recipients, yet its clinical significance and optimal management remain controversial (69). For ASB in kidney transplant recipients, clinical management should be individualized based on multiple factors, including time since transplantation, graft function, degree of immunosuppression, presence of urinary devices, local antimicrobial resistance patterns, and institutional protocols (36, 70). In clinical management, this translates into considerable heterogeneity in practice across centers. While some centers may consider empirical antibiotic therapy for early post-transplant ASB (e.g., within the first month) in high-risk patients (71), others may adopt a more conservative approach, closely monitoring urine cultures and renal function to avoid unnecessary antibiotic exposure and development of resistance (70). In clinical practice, there is no standardized protocol for the frequency of screening urine cultures, because controlled evidence defining an optimal interval is lacking (70); instead, the interval is individualized according to the time since transplantation, individual risk factors, and institutional protocols, with reported schedules ranging from weekly testing in the early post-transplant period to quarterly testing thereafter (72).

Symptomatic Post-KT UTIs may include UTIs confined to the bladder and lower urinary tract, presenting with typical bladder irritation symptoms, abnormal micturition (dysuria, burning sensation), mild suprapubic pain, as well as mild or transient elevation of serum creatinine levels (43). Severe cases may involve upper UTIs, such as acute graft pyelonephritis (AGPN) (71), and can further develop into urosepsis. Beyond systemic toxic manifestations, they may also present with classic yet abnormally positioned low back pain and gastrointestinal symptoms, resulting in misdiagnosis (36, 70). Furthermore, recurrent episodes are also common (64). In summary, the clinical manifestations of Post-KT UTIs are diverse, making a clear diagnosis difficult through routine physical examinations. These categories are summarized in **Figure 1**.

**Figure 1:**
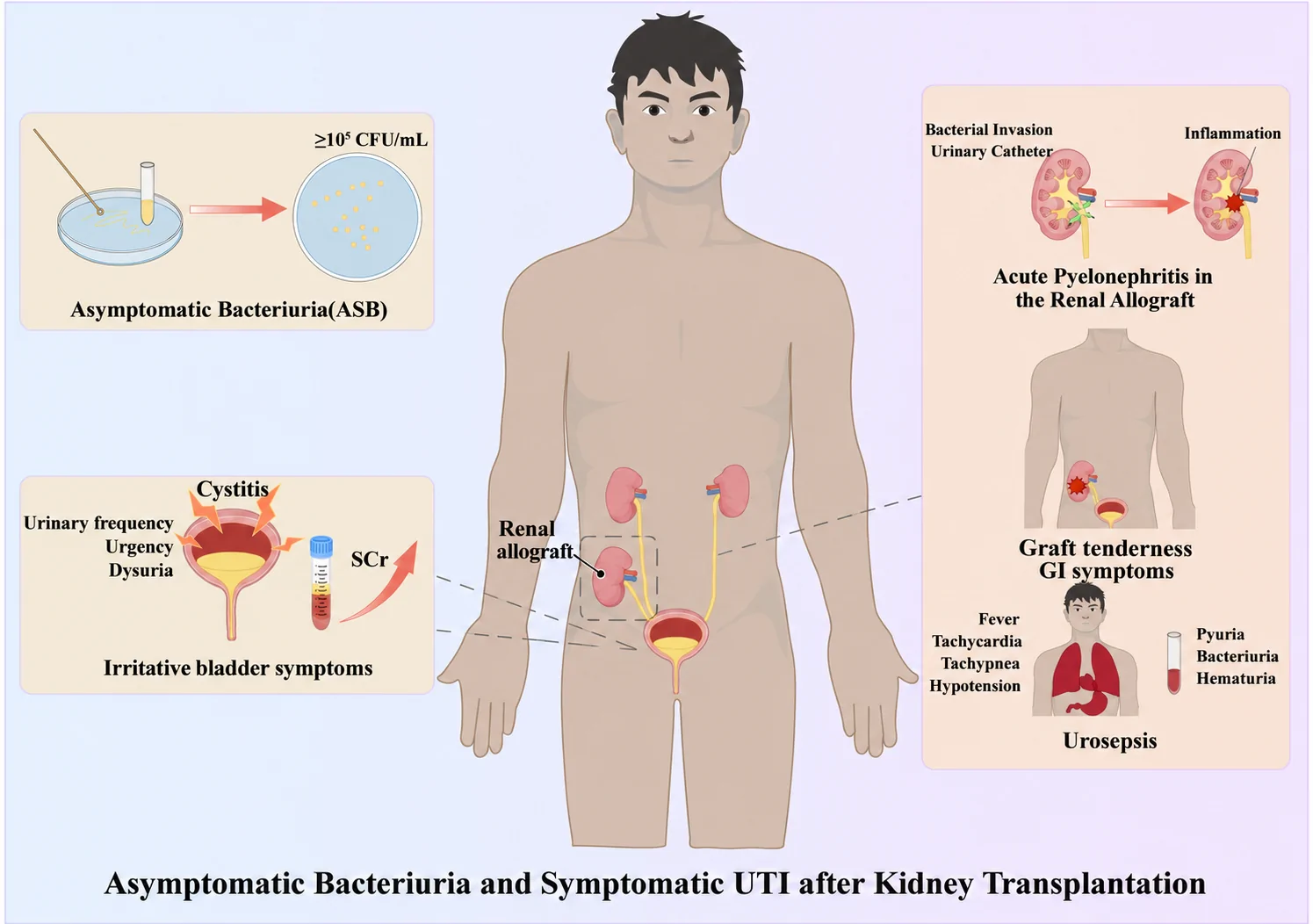
A variety of clinical presentations of post-kidney transplant UTIs.

## 5 Diagnostic approaches for post-KT UTIs

Given the nonspecific clinical manifestations described above, auxiliary examinations are essential for the diagnosis of Post-KT UTIs in kidney transplant recipients (36). Diagnostic evidence is generally provided through laboratory tests such as urinalysis, urine culture, serum biomarkers, and molecular diagnostics (43). In addition, imaging examinations can be used to assess the severity of Post-KT UTIs and provide a reference basis for percutaneous drainage or surgical intervention (64).

### 5.1 Traditional laboratory examinations

For kidney transplant recipients with Post-KT UTIs, urinalysis and urine microscopic examination are important methods for the preliminary screening of Post-KT UTIs (36, 43). Among the indicators, positive leukocyte esterase can support the diagnosis of UTIs. However, the test is more sensitive than specific (73), and its positive predictive value varies considerably between patient populations (74). A negative result therefore cannot completely rule out the presence of UTIs, because the leukocyte response may be weakened due to the use of immunosuppressants in kidney transplant patients (36, 70). Additionally, nitrite offers some referential value for UTI diagnosis, yet it is only effective against Enterobacteriaceae (e.g., *E. coli*) capable of reducing nitrate to nitrite (21, 43). Therefore, a negative nitrite result does not allow for the exclusion of UTIs. If bacteria or leukocytes (≥5–10/HPF) are observed in urinary sediment under a microscope, both can provide indirect evidence for the occurrence of UTIs (43, 73). However, due to factors such as susceptibility of specimens to contamination and mild pyuria caused by the transplanted kidney itself or body rejection reactions, the aforementioned tests can hardly provide definitive evidence for the diagnosis of Post-KT UTIs (36, 43, 70).

Urine culture remains the gold standard for the microbiological diagnosis of post-KT UTIs. The American Society of Transplantation (AST) Infectious Diseases Community of Practice guidelines specify syndrome-specific colony count thresholds for a uropathogen in midstream urine. These are >10³ CFU/mL for acute simple cystitis and >10^4^ CFU/mL for acute pyelonephritis or complicated UTI. Pyuria (>10 WBC/mm³) usually accompanies both syndromes, but its absence does not exclude UTI when the clinical presentation is otherwise consistent (36). In the absence of urinary or systemic symptoms attributable to infection, >10^5^ CFU/mL is classified as asymptomatic bacteriuria, which should be distinguished from UTI. Lower colony counts may still be clinically relevant in symptomatic patients, although evidence for low-count cystitis is derived mainly from Escherichia coli infections (36). If pyelonephritis or urosepsis is suspected, blood cultures should also be obtained before antimicrobial therapy to assess for bacteremia and guide treatment (36). Furthermore, in kidney transplant patients with systemic toxic manifestations and urosepsis, blood cultures are necessary to determine the specific pathogenic microorganisms responsible for the UTIs and inform antibiotic treatment (75).

### 5.2 Novel technologies in laboratory examinations

Conventional urine culture, although the reference standard, does not identify a causative pathogen in a considerable proportion of kidney transplant recipients with suspected UTI. Culture-negative presentations arise when organisms are fastidious or present at low loads, or when prior antimicrobial exposure suppresses growth, and they constitute a key diagnostic challenge. To address these diagnostic challenges, novel biomarkers and molecular detection technologies have been investigated as complementary approaches, particularly in cases of suspected UTI with negative conventional urine culture results.

Beyond C-reactive protein, which is used to dynamically monitor treatment response in kidney transplant recipients, recent research has established the diagnostic utility of other markers, including IL-6 released from bacterially-invaded urothelial cells and procalcitonin (PCT), which is strongly correlated with bacterial infection, facilitated by the discovery of new biomarkers and improved detection methods (76). A study by Gill indicated that the combined detection of urinary ATP concentration and bacteria in shed urothelial cells may be a sensitive detection method for occult infections in kidney transplant recipients (77).

Precision molecular detection is also a rapidly developing laboratory diagnostic method in recent years. Clinically, polymerase chain reaction (PCR) is used for the accurate identification of pathogens causing Post-KT UTIs. It has a relatively obvious advantage over traditional bacterial culture methods in the detection rate of fastidious bacteria (such as *U. urealyticum*). However, traditional PCR technology still has disadvantages such as long time consumption and high requirements for specimens (78). As an emerging detection technology that relies on high-throughput sequencing platforms to simultaneously perform parallel sequencing of millions of microbial DNA fragments in urine, mNGS demonstrates certain diagnostic advantages in the diagnosis of Post-KT UTIs. In a small retrospective case series of ten non-transplant patients with acute UTI (four with urosepsis), 16S rRNA amplicon sequencing detected culture-negative potentially pathogenic species, including pathogens identified in blood by sequencing when blood culture was negative (79), and mNGS can also detect UTIs caused by viral sources that are difficult to identify via traditional culture methods (80). A systematic review and meta-analysis of 17 studies reported a significantly higher pathogen detection rate for mNGS than for culture (OR 2.87), with pooled sensitivity of 0.89 and AUROC of 0.89, although the certainty of evidence remains low (81). Notably, studies conducted specifically in kidney transplant recipients have shown similar advantages (81), and mNGS has also been applied to infection diagnosis after liver transplantation (82).

Considering the limitations of mNGS (high cost), PCR (prolonged testing time), and current biomarkers (insufficient specificity), a microfluidic inertial impedance cytometer-based detection technology has recently been developed. Utilizing the inertial focusing effect based on cell size, it enables enhanced analysis and screening of neutrophils in urine within a short timeframe (< 5 minutes), aiming to identify UTI-positive patients (83). It has been observed that CRISPR technology has been extensively studied for pathogen infection diagnosis. For instance, the ERA/CRISPR-Cas12a dual system — constructed by integrating the high specificity and endonuclease activity of the LbCas12a protein with enzyme recombination amplification technology — has been employed for *Mycoplasma* detection. In head-to-head comparisons for M. pneumoniae detection, the ERA/CRISPR-Cas12a system required less time and lower cost than quantitative PCR (qPCR), while maintaining comparable specificity (84). In one study, a LbuCas13a-based CRISPR detection kit detected RNA from urinary tract pathogens in less than 1 hour, with a lower limit of detection (LOD) of 10^6^ copies/μL and no requirement for pre-amplification. If validated in larger cohorts, such assays may provide more rapid results to inform clinical decision-making (85). Meanwhile, the study pointed out that an RNase inhibitor has been added to the LbuCas13a-based CRISPR kit to address the high RNase content in urine specimens and prevent the degradation of target RNA. However, LbuCas13a has low sensitivity. Isothermal CRISPR-based amplification has been used to detect BKV and JCV and to provide absolute quantification (86). Future studies will be needed to determine whether room-temperature amplification technologies such as ERA can improve the sensitivity of CRISPR-based assays for UTI diagnosis, given that ERA has thus far been validated only in non-urinary pathogen models (84) (**Figure 2**).

**Figure 2:**
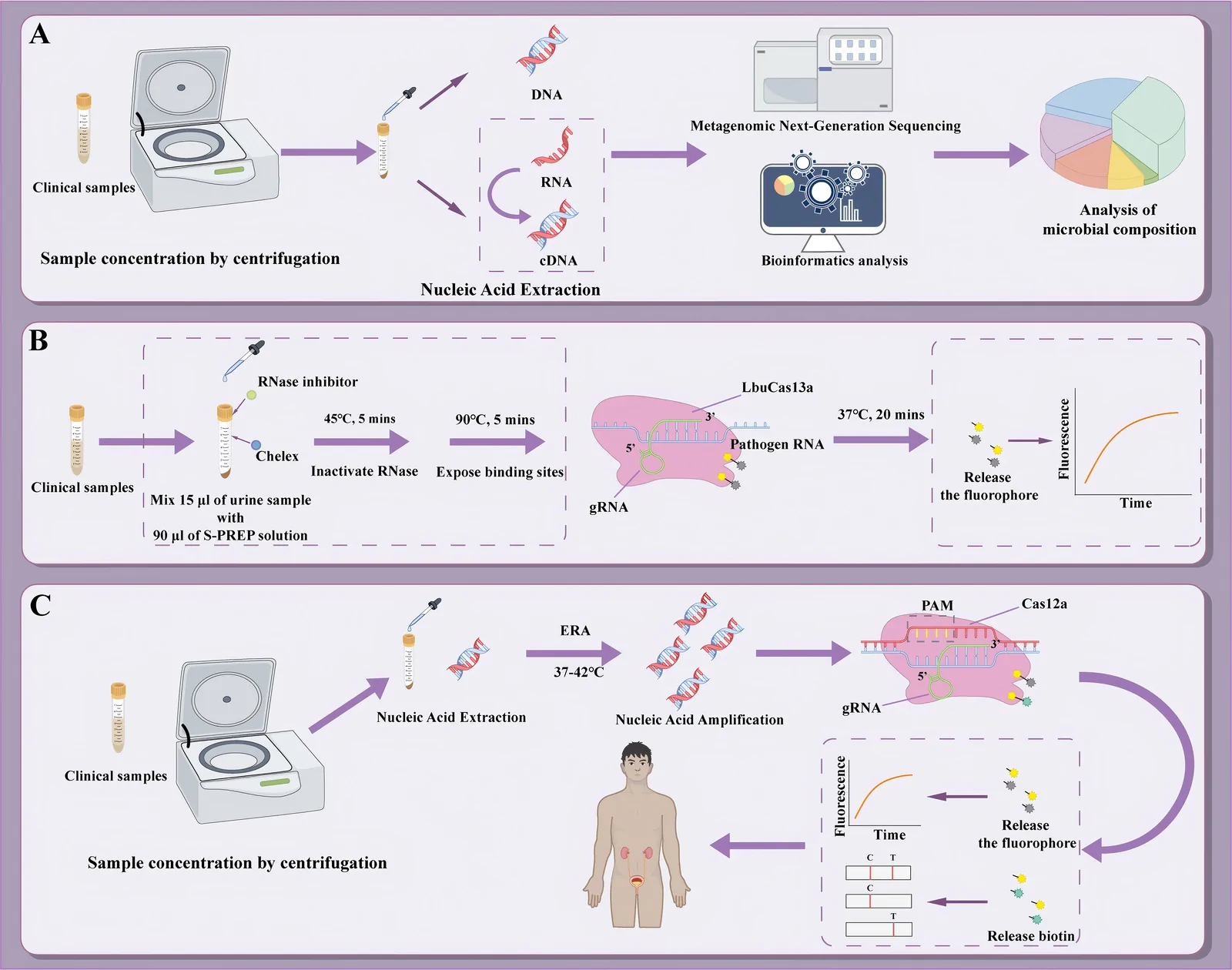
**(A)** mNGS workflow schematic; **(B)** CRISPR-LbuCas13a workflow schematic; **(C)** ERA/CRISPR-Cas12a dual system workflow schematic. The schematic is adapted from the CRISPR-based detection workflow for *Mycoplasma pneumoniae* (84).

In addition to biomarkers and molecular diagnostics, a microfabricated electrochemical DNA biosensor achieved species-specific detection of uropathogens in urine within 45 min in a small feasibility study. The arrays lyse bacteria to release 16S rRNA, hybridize it with species-specific probes, and catalyze 3,3′,5,5′-tetramethylbenzidine (TMB) reaction on the sensor surface, producing an electrical signal that enables rapid identification (87), although its performance in kidney transplant recipients has not been evaluated. Considering the lack of equipment and infrastructure in low- and middle-income countries, the development of point-of-care test (POCT) strips based on chromogenic bacterial culture media may also be one of the main application directions in the future (88).

#### 5.2.1 Limitations of novel diagnostic technologies

Currently, novel technologies such as mNGS and CRISPR still face prominent clinical application challenges, which restrict their large-scale popularization in transplant centers, especially primary-level institutions. One major barrier is the high cost of testing. Compared with conventional urine culture, mNGS costs 5–10 times as much, which is a burden that many patients in low- and middle-income countries (LMICs) cannot afford (89). Stringent laboratory requirements represent another important barrier to the large-scale implementation of emerging technologies. For example, precise detection by CRISPR-based assays requires trained technical personnel and standardized experimental platforms for quality control, resources that are not yet available in most primary-level transplant centers (90). In addition, most existing studies of these emerging technologies are small, single-center, retrospective investigations, resulting in insufficient clinical validation and a lack of universally accepted quantitative thresholds for result interpretation (81, 91). Another barrier to the widespread adoption of these emerging technologies is the difficulty of bioinformatic interpretation, which arises from the large volume of mNGS sequencing data and the lack of standardized analysis pipelines and reporting criteria. This may lead to false-positive results (e.g., due to environmental contamination) and false-negative results (92, 93).

#### 5.2.2 Comparative analysis of traditional and novel diagnostic technologies for post-KT UTIs

To provide a clear clinical reference for the selection of detection technologies, the core characteristics of traditional and novel diagnostic technologies for Post-KT UTIs are compared from three key dimensions (**Table 3**). Traditional urine culture remains the “gold standard” for Post-KT UTIs diagnosis due to its low cost and good clinical applicability; emerging technologies such as mNGS, CRISPR-based assays, microfluidic cytometry, and biosensors serve complementary roles in selected cases. Technology selection should follow the clinical syndrome, pre-test probability, turnaround needs, and laboratory capacity.

**Table 3:** Comparative analysis of diagnostic technologies for post-KT UTIs.

Detection technology	Detection speed	Pathogen detection range	Clinical application scenario	Sources
Traditional urine culture	24–48 h	Common bacteria, fungi	Routine Post-KT UTIs; Efficacy verification after empirical antibiotic treatment	(36, 37)
Conventional PCR	4–6 h	Common bacteria, mycoplasma, partial viruses	Targeted adjunct for fastidious or predefined pathogens	(53, 78)
mNGS	12–24 h	Bacteria, fungi, viruses, mycoplasma, mixed pathogens	Occult/complex mixed Post-KT UTIs; Atypical viral-induced Post-KT UTIs; Refractory Post-KT UTIs	(90, 91, 94, 95)
CRISPR-based assay	< 1 h	Specific target pathogens (BKV/JCV/Mycoplasma)	POCT for Post-KT UTIs; Rapid screening of high-risk pathogens; Bedside emergency diagnosis	(85, 86, 96, 97)
Microelectrochemical sensor array	1–2 h	Common uropathogenic bacteria	Rapid preliminary screening of Post-KT UTIs in primary institutions	(87, 88)

Turnaround times vary by assay and laboratory workflow.

#### 5.2.3 Clinical integration of conventional and emerging diagnostic technologies

Clinical integration combines molecular pathogen detection with clinical assessment, urine culture, and antimicrobial susceptibility testing (36, 37, 90). In kidney transplant recipients with suspected symptomatic UTI, diagnosis should remain syndrome-based and incorporate clinical presentation, urinalysis, urine culture, prior microbiology, antimicrobial exposure, urinary devices, and graft function. Urine culture remains the reference method because it provides organism identification and phenotypic antimicrobial susceptibility testing. In clinically stable recipients with lower-tract symptoms and a plausible uropathogen identified by culture, these data are usually sufficient to guide targeted treatment. Blood cultures should be obtained when graft pyelonephritis, systemic toxicity, or urosepsis is suspected, as bloodstream infection may affect antimicrobial selection and duration (36, 37).

Targeted nucleic-acid amplification tests may be useful when symptoms or pyuria persist despite negative cultures, particularly after antimicrobial exposure or when fastidious organisms are suspected. Culture-independent studies in renal transplant recipients with sterile pyuria have detected *Ureaplasma* spp., *Mycoplasma hominis*, and other organisms that may be missed by routine culture, supporting selective rather than universal PCR use (53). However, PCR and sequencing may also detect colonizing, nonviable, or clinically irrelevant organisms; results should therefore be interpreted alongside symptoms, organism burden, specimen quality, conventional microbiology, and treatment history (90, 94).

mNGS extends pathogen detection in selected cases of recurrent or persistent infection, including culture-negative presentations (90, 94, 95). In a study of 19 kidney transplant recipients with recurrent UTI, mNGS detected additional bacterial, fungal, and viral signals compared with culture (91). However, interpretation should integrate these findings with clinical presentation, specimen quality, and culture-based susceptibility testing (90, 94).

## 6 Clinical therapy and prophylaxis of post-KT UTIs

As a key initial step in managing post-KT UTI, empirical antibiotic therapy should be initiated promptly in patients with suspected symptomatic UTI, particularly those with severe presentations or high-risk features, guided by local antimicrobial susceptibility patterns and prior culture data, and subsequently de-escalated to targeted agents once antimicrobial susceptibility results become available. Empirical antibiotic therapy for post-KT UTI should be individualized according to syndrome severity and guided by current guidelines. Kidney Disease: Improving Global Outcomes (KDIGO) recommends hospitalization with intravenous antibiotics for allograft pyelonephritis (98). European Association of Urology (EAU) guidelines favor fluoroquinolones and cephalosporins as first-line agents for empirical treatment of pyelonephritis. When urosepsis is suspected, empirical antimicrobial coverage for extended-spectrum β-lactamase (ESBL)-producing organisms is warranted (99). Empirical regimens should be tailored to local antimicrobial susceptibility data, the patient’s prior culture results and colonization history, and potential drug–drug interactions with immunosuppressive agents.

Due to the broad spectrum of potential uropathogens, empirical antibiotic therapy for post-KT UTI should provide coverage against gram-negative bacilli (including *Pseudomonas aeruginosa* in severe cases) and gram-positive cocci, including enterococci (100). For hospitalized patients with systemic infection, parenteral third- or fourth-generation cephalosporins, piperacillin-tazobactam, or carbapenems are commonly used; aminoglycosides should be employed cautiously given their nephrotoxicity in allograft recipients, and fluoroquinolones are generally discouraged as empirical agents owing to rising resistance rates and their interactions with calcineurin inhibitors (43, 99). Furthermore, Trimethoprim-sulfamethoxazole (TMP-SMX) warrants particular emphasis because of its dual role in this population. As recommended by KDIGO, daily TMP-SMX for at least first 6 months post-transplant serves not only as Pneumocystis pneumonia prophylaxis but also reduces the incidence of UTI and related bacteremia (98). However, prolonged antimicrobial exposure may promote the selection of drug-resistant organisms, including ESBL-producing Enterobacteriaceae, underscoring the importance of surveillance-driven adjustments to both prophylactic and therapeutic strategies (100, 101). After the causative pathogens of Post-KT UTIs are identified, the treatment plan should be adjusted according to antimicrobial susceptibility testing results to prevent the development of antimicrobial resistance. For patients with fungal urinary tract obstruction, surgical intervention or percutaneous urinary drainage may be required (102).

Preoperative antimicrobial therapy for KT, rational use of immunosuppressants, and regular monitoring of urinary catheters are effective measures to prevent Post-KT UTIs (103, 104). In view of the role of the gut-urinary microbiota axis in the occurrence of Post-KT UTIs, modulation of the intestinal and urinary microbiota has been proposed as a potential preventive strategy (28), although interventional evidence in kidney transplant recipients remains lacking. In addition, CRISPR technology has been proven to effectively reduce the formation of biofilms on urinary catheters (105); however, these findings are derived from gene-knockout experiments in a laboratory strain, and clinical applicability remains to be established. Currently, several novel preventive and therapeutic strategies for Post-KT UTIs are under investigation, including: (1) CRISPR-based approaches to disrupt biofilm formation on urinary catheters, which remain at the preclinical stage but hold promise for reducing catheter-associated urinary tract infections (105, 106); (2) immune monitoring–guided personalized adjustment of immunosuppressive regimens to balance the risks of rejection and infection (107, 108); (3) CRISPR-Cas3–enhanced bacteriophage therapy (e.g., LBP-EC01), which has shown safety and proof of mechanism in a phase 1b trial of E. coli urinary colonization and may offer an option for antibiotic-resistant infections (109). The preliminary results of these studies show good clinical application potential, but large-sample, multi-center clinical verification is still needed.

## 7 Conclusions

In summary, Post-KT UTIs are caused by the interaction of multiple risk factors including immune dysfunction, anatomical abnormality, and microbial imbalance, with the characteristics of diverse pathogens, atypical clinical manifestations, and easy development of drug resistance. Their clinical management should adhere to the principle of “combination of prevention and treatment”, and integrate risk factor intervention, precise pathogen diagnosis and individualized treatment, while balancing the anti-infection effect and the prevention of graft rejection. The diagnosis of post-KT UTI remains primarily based on urine culture, which continues to serve as the reference standard; emerging technologies such as mNGS and CRISPR-based assays have shown promise in preliminary studies, particularly for culture-negative and polymicrobial infections, although they currently function as adjuncts to conventional culture rather than replacements. With the optimization of technology (e.g., cost reduction, sensitivity improvement) and the accumulation of clinical validation evidence, these novel technologies are expected to complement the traditional diagnostic system and become an important part of the precision diagnosis of Post-KT UTIs. In terms of treatment, the management of Post-KT UTIs will gradually shift from empirical antibiotic use to precision and individualized treatment based on pathogen detection and drug resistance gene analysis. Looking ahead, an integrated strategy combining modulation of the gut–urinary microbiota axis with molecular diagnostics, biomarker monitoring and, potentially, multi-omics approaches (metabolomics, transcriptomics) could, if validated, support earlier pathogen identification, more targeted intervention and longitudinal monitoring of post-KT UTI.
